# Developing a specialist children’s nursing workforce in sub-Saharan Africa: a descriptive programme evaluation

**DOI:** 10.1186/s12912-020-00502-1

**Published:** 2020-12-01

**Authors:** Jennifer Ruthe, Natasha North

**Affiliations:** 1 Humanitarian and Conflict Response Institute, Ellen Wilkinson Building, University of Manchester, Oxford Road, Manchester, UK; 2 The Harry Crossley Children’s Nursing Development Unit, Department of Paediatrics and Child Health, University of Cape Town, Red Cross War Memorial Children’s Hospital, Klipfontein Road, Rondebosch, Cape Town, South Africa

**Keywords:** Education, Nursing education research, Nursing, Nurses, Advanced practice nursing, Critical care nursing, Specialities, Students, Universal health coverage

## Abstract

**Background:**

Achieving Universal Health Coverage in low and lower-middle income countries requires an estimated additional five and a quarter million nurses. Despite an increasing focus on specialist nursing workforce development, the specialist children’s workforce in most African countries falls well below recommended densities. The Child Nursing Practice Development Initiative was established with the aim of building the children’s nursing workforce in Southern and Eastern Africa, and Ghana. The purpose of this evaluation was to enable scrutiny of programme activities conducted between 2008 and 2018 to inform programme review and where possible to identify wider lessons of potential interest in relation to specialist nursing workforce strengthening initiatives.

**Methods:**

The study took the form of a descriptive programme evaluation. Data analysed included quantitative programme data and contextual information from documentary sources. Anonymised programme data covering student enrolments between January 2008 and December 2018 were analysed. Findings were member-checked for accuracy.

**Results:**

The programme recorded 348 enrolments in 11 years, with 75% of students coming from South Africa and 25% from other sub-Saharan African countries. With a course completion rate of 94, 99% of known alumni were still working in Africa at the end of 2018. Most graduates were located at top-tier (specialist) public hospital facilities. Nine percent of known alumni were found to be working in education, with 54% of graduates at centres that offer or plan to offer children’s nursing education.

**Conclusion:**

The programme has made a quantifiable, positive and sustained contribution to the capacity of the specialist clinical and educational children’s nursing workforce in nine African countries. Data suggest there may be promising approaches within programme design and delivery in relation to very high course completion rates and the retention of graduates in service which merit further consideration. Outputs from this single programme are however modest when compared to the scale of need. Greater clarity around the vision and role of specialist children’s nurses and costed plans for workforce development are needed for investment in specialist children’s nursing education to realise its potential in relation to achievement of Universal Health Coverage.

## Background

It is widely acknowledged that a significant increase in the size of the nursing workforce is needed in order for African countries to make progress towards achieving Universal Health Coverage (UHC) [1, 2]. Half the world’s population live in countries that are, collectively, served by 20% of the world’s nurses [3]. The global shortage of nurses is estimated at 5.9 m, with 5.3 m (89%) needed in low and lower middle-income countries, where the growth in the number of nurses is often barely in line with population growth [3]. This makes achieving the Sustainable Development Goal targets for reduced child mortality and morbidity, and increased wellbeing of children, particularly challenging [4].

One quarter of all the world’s children – an estimated 580 million - live in African nations [5, 6], where specialised care from appropriately skilled and qualified nurses can be key to the survival of the sickest [7-10]. But the specialist paediatric health workforce is far below recommended densities in most African countries [8, 10, 11], with children’s nurses (defined as registered nurses with an additional specialist qualification in paediatric nursing) forming barely 1% of the nursing workforce [12].

The UN-led High-Level Commission on Health Employment and Economic Growth advocated significant scaling up of transformative, high-quality education for health workers [1]. Specifically in relation to nursing, the inaugural State of the World’s Nursing report has called for a massive acceleration in nurse training to address global needs [3]. A number of African countries are working to strengthen the capacity of their paediatric workforces, with support from local and international partners [13, 14]. There is some evidence of recent increases in the training output of children’s nurses in a number of African countries through new educational programmes [12] but concerns persist about retention of trainees through international migration, public-private drift, and ultimately whether any growth achieved can be sustained [15, 16].

Despite calls for investment in health worker training to be supported by instructive monitoring and evaluation [17, 18], there remain few published evaluations of African-based nurse education programmes. Qualitative evaluations predominate [19-22]. While the WHO and others advise that education programme evaluation should include longer-term follow up and consider not only graduate output, but also the situation of graduates post-training [17, 18], published evaluations commonly focus on the activity of a single institution, a short period of programme activity, and/or the experiences of a single student year group. With rare exceptions [23], few published evaluations present data analysing student throughput or demographics in-depth. Longer-term programme impacts remain under-explored, which limits opportunities to learn lessons about educational programmes seeking to support nursing workforce strengthening.

## Methods

### Aims

The purpose of the evaluation was to enable scrutiny of programme activities conducted between 2008 and 2018 by the Child Nursing Practice Development Initiative (CNPDI) at the University of Cape Town, to inform programme review and where possible to draw wider lessons in the context of the needs and challenges described above. A description of the programme is provided below (see Setting). Operational commitments meant the programme had not previously conducted a formal programme evaluation, and had a database holding 11 years of data yet to be explored in detail. This included limited follow up information about graduate situations post-training.

In relation to the policy-making and decision-making context for the evaluation [24], the evaluation questions that the CNPDI programme team initially wished to understand related to three main areas: demand for training; throughput of trainees; and the location and utilisation of graduates.

The aim of the study therefore, was to generate an overview of training activity and student throughput attributable to CNDPI programmatic activities between 2008 and 2018, in order to allow consideration of the three evaluation issues identified.

Objectives were to:

1. Identify appropriate data sources to enable examination of the evaluation issues.
2. Present numerical and visual representations of these data relating to:
  training activity and throughput (student enrolment, course completions)
  employment status and practice environment of graduates.
3. Generate recommendations for further programme monitoring and evaluation and note implications for wider learning of relevance to programmes seeking to strengthen the specialist nursing workforce in Africa.

### Design

The study took the form of a descriptive programme evaluation. Multiple methods were used. The majority of evaluation activities involved analysis of secondary quantitative data from two sources (see ‘Processes’). Member checking [25, 26], usually applied within qualitative research, was used as a complementary method because of the importance of accurately understanding the context in which the programme was operating [27].

The overall design of the study and selection of methods was informed by the evaluation questions and context articulated by the CNPDI programme team in dialogue with the researcher [24]. A brief narrative programme description, including logic model [28], and a summary of research processes are provided in the sections that follow.

### Setting

The CNPDI is a nurse-led academic programme based in the Department of Paediatrics and Child Health at the University of Cape Town, South Africa. In 2006, the Initiative was tasked with re-establishing children’s nursing training at the University. Subsequent donor funding agreements mandated the programme to provide education and capacity building intended to lead to the creation of new education programmes in targeted countries. These countries have to date included Malawi, Botswana, Namibia, Zambia, Zimbabwe, Uganda, Kenya and Ghana. Children’s nursing education programmes at the University of Cape Town are offered collaboratively under the governance of the Division of Nursing and Midwifery as the accredited School of Nursing. Educational programmes offered at the University of Cape Town include a one-year Post-Graduate Diploma in Child Nursing (PGDip-CN); a one-year Post-Graduate Diploma in Critical Care Child Nursing (PGDip-CCCN); and a two-year professional Master of Nursing in Child Nursing programme (MNCN) (commenced 2016).

The programme developed a Theory of Change shortly prior to the design of this evaluation study, which provided the logic model for the evaluation [29]. CNPDI’s stated long-term goal is a strengthened children’s nursing workforce, working to best possible effect in Africa’s health care systems. The anticipated outcome is that this will indirectly contribute to progress towards UHC and lead to improved infant and child health outcomes in Africa.

At the time the programme commenced activities, South Africa was the only country in the region providing specialist children’s nursing training. Across South Africa as a whole, in 2018 there were reported to be seven training institutions offering 11 different programmes and producing approximately 180 children’s nurses per year [12]. Regulatory changes involving the phasing out of “legacy qualifications” are likely to have reduced the number of training providers from 2019, but the extent of this reduction is not yet known [30]. During the period of study the University of Cape Town was the only provider of critical care children’s nursing education in South Africa and one of only two providers serving southern and eastern Africa. Information regarding the extent of international enrolments for children’s nursing training programmes at South African universities is not available, but it is believed that international enrolments have declined. In recent years the University of Cape Town has been the only South African university training children’s nursing students from other African countries [12, 31].

### Participants

The study mainly involved secondary data analysis of quantitative information. Two CNPDI team members (the Programme Manager and the Research Programme Director) participated in member checking [25, 26] of data presentation to ensure accuracy, and commented on emerging findings.

### Processes

Evaluation research processes are described following the reporting headings recommended by Clarke [32] in relation to: data sources; indicator selection; data analysis and data presentation.

### Data sources

Two sources of data were defined, in relation to programme information and contextual information. For programme information, the programme already maintained a database which routinely recorded information about training activity. It was determined that critical examination and analysis of this information, under approved conditions to ensure ethical research conduct and compliance with data protection requirements, would be a suitable method through which to acquire the majority of data relevant to the pursuit of the study aims and evaluation questions. In relation to contextual information, it was determined that publicly available information from documentary sources including government websites, national data sets, institutional websites and online academic placement finders would be used to enable consideration of programme activity in context, for example the type and sector of health facilities in a given country.

### Indicator selection

The evaluation questions described above were used to structure the selection of indicators against which data would be analysed. The programme database included information on all student enrolments over the last 11 years by age, gender, nationality, year of registration, course selection, funding source and graduation date. The WHO’s National Health Workforce Accounts (NHWA) [33] minimum data set recommendations were also consulted as a reference to inform the selection of indicators. This process led to the inclusion of five additional fields, with indicators then refined by the introduction of a threshold completion rate (> 80%) to ensure that analysed data would present an accurate picture of programme throughput. This led to the exclusion of a number of fields, as shown in Table 1. Table 1 shows the data fields initially identified through the programme database, and the final set of indicators that were included after appraisal, with sources.

**Table 1:** Indicator selection process

	Research Indicator	Source	Completion	NHWA indicator [33]
INPUTS	Enrolment numbers	Programme Information	100%	1.00
	[Age]^a^	Programme Information	Insufficient data	1.03
	Gender	Programme Information	99%	1.04
	Referral country	Programme Information	100%	1.08
	Programme of study	Programme Information	100%	8.06
	Base institution	Programme Information	97%	8.01
	Location^a^	Contextual Information	89%	1.07, 1.08
	Country^a^	Contextual Information	Based on above	1.07, 1.08
	Sub-national^a^	Contextual Information	Based on above	1.02
	Facility type^a^	Contextual Information	Based on above	1.06, 8.01
	Institution sector^a^	Contextual Information	Based on above	1.05
	Health system level^a^	Contextual Information	Based on above	8.01
	[Years of experience]	Programme Information	Insufficient data	
	[Year registered]	Programme Information	Insufficient data	1.01
OUTPUTS	Year completed	Programme Information	100%	2.03, 2.07, 2.08
	Graduation rate	Programme Information	Based on above	2.03, 2.07, 2.08
	Graduate skill-mix	Programme Information	Based on above	8.06
	[Returned home]	Programme Information	Insufficient data	1.00, 5.01
	[Returning institution]^a^	Programme Information	Insufficient data	1.02, 5.02, 5.04, 5.05, 5.06
	[Location]^a^	Contextual Information	Based on above	1.07, 1.08
	[Country]^a^		Based on above	
	[Sub-national]^a^		Based on above	
	[Facility type]^a^	Contextual Information	Based on above	1.05, 1.06, 8.01
	[Institution sector]^a^	Contextual Information	Based on above	1.05, 1.06
	[Health system level]^a^	Contextual Information	Based on above	8.01
	[Returning role]	Contextual Information	Insufficient data	8.06
OUTCOME	Current institution	Programme Information	87%	1.01, 1.07, 1.08
	Location^a^	Programme Information	Based on above	1.07, 1.08
	Country^a^	Contextual Information	Based on above	1.07, 1.08
	Sub-national^a^	Contextual Information	Based on above	1.02
	Facility type^a^	Contextual Information	Based on above	1.05, 1.06, 8.01
	Institution sector^a^	Contextual Information	Based on above	1.05, 1.06
	Health system level^a^	Contextual Information	Based on above	8.01
	[Current role]	Programme Information	Insufficient data	8.06

^a^ Indicator added to study based on assessment of NHWA indicators

[X] Indicator removed from study due to insufficient data

To structure the analysis of the publicly available contextual information, a common data set was constructed for each country identified in the programme’s database, depicting the tiered structure of each national health system and recording the number of facilities at each tier. This was compiled through structured searching of publicly available documentary information including government health strategies, which were available for all countries in the study.

### Member checking

Member checking [25, 26] was used to check the congruence of analysed data with programme team members’ experiences [34]. Corroboration through member checking also assisted the researcher in gaining deeper understanding of the wider programme context and specific situations [27, 35]. The rationale for using member checking aligned with Boaz et. al’s recommendations for stakeholder engagement in research, with the intention of leading to co-creation of accurate and practically useful information [36]. The two programme team members were given drafts of the findings on two occasions and asked to verify their accuracy, as well as to challenge, clarify or elaborate on emerging interpretations of the data. They were also provided with a draft of the near final report and asked to comment on and verify the findings and interpretations. Member checking took the form of written comments and telephonic conversations, after which amendments were made to the draft.

### Data analysis

Database reports comprising anonymised structured quantitative programme data as specified in Table 1 were generated by CNPDI programme staff and made available to the researcher. Data analysed covered all student enrolments between January 2008 and December 2018 including age, gender, nationality, year of registration, educational programme, funding and graduation date. Analysis of data was conducted using Excel, with geographical locations of facilities plotted using Google Maps.

### Data presentation

Data were presented in the form of numerical reports for all indicators. Data visualisations were produced for each of the three evaluation questions, showing geographic and demographic data. A comprehensive report was produced and stored in an online repository with a persistent link [37].

### Reliability and validity

Reliability and validity were supported by careful selection of indicators and the imposition of an 80% field completion threshold. Member checking and corroboration of data with programme staff supported greater accuracy of data presentation and interpretation [25, 26, 34]. Availability of data from an 11 year period supported evaluation of programme outputs and outcomes beyond the immediate term [17, 18].

## Results

Data are presented in relation to the aims of the study as follows:

- Training activity and throughput (student enrolment, demand for training, course completions).
- Geographical mapping of graduates by country, region and facility, showing reported employment status and practice environment.

### Training activity and throughput

#### Student enrolment

**Fig. 1:**
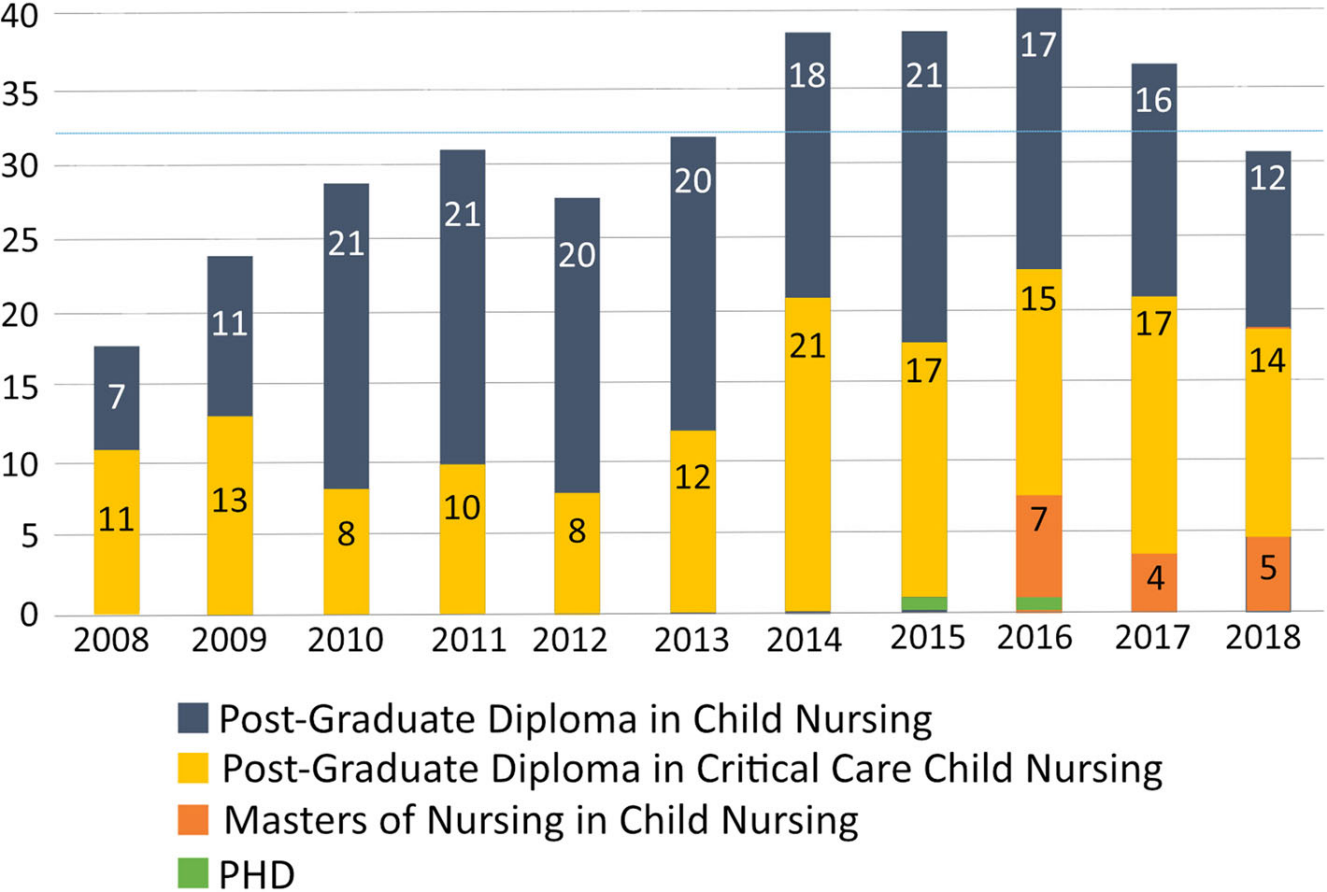
Demand for training by year and educational programme

**Fig. 2:**
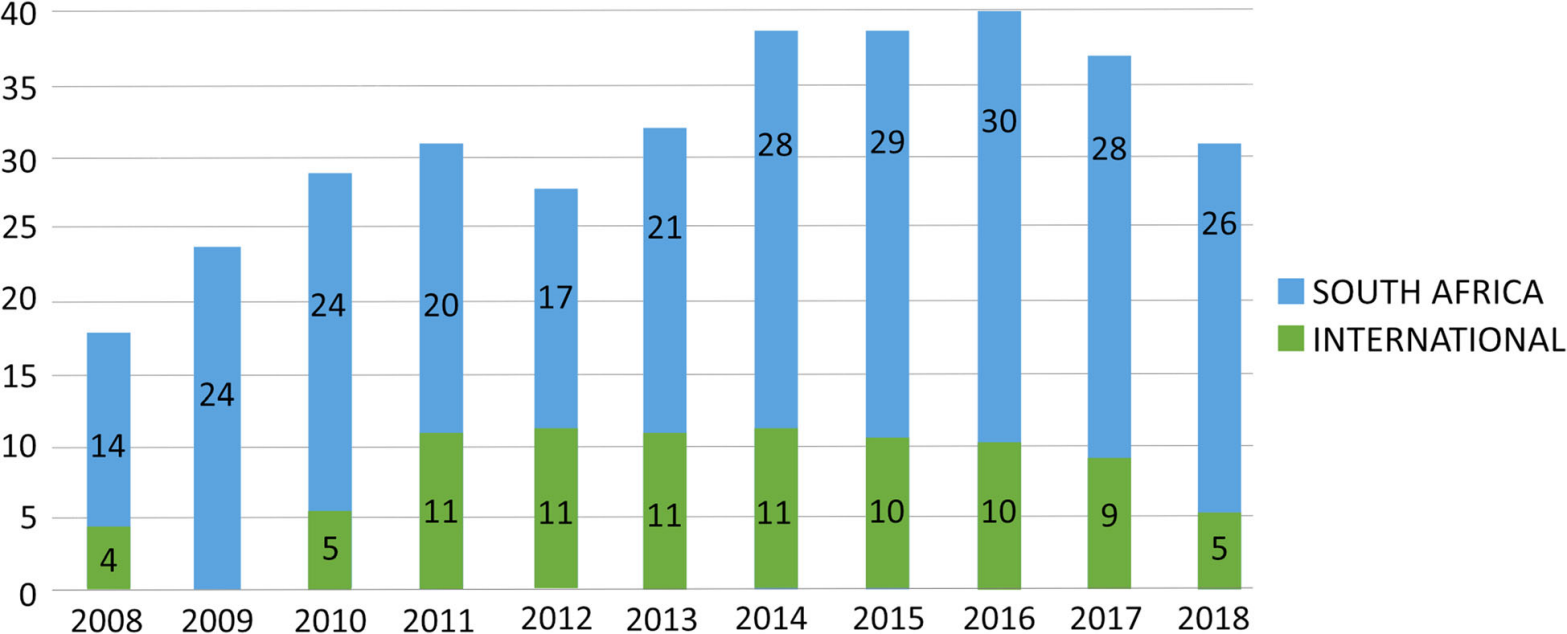
Demand for training by year and location

Analysis of programme data revealed that the CNPDI processed a total of 348 enrolments between January 2008 and December 2018 (see Fig. 1). Of these, 75% (261) were from South Africa and 25% (87) travelled from 10 other sub-Saharan African countries (see Fig. 2). Malawi (33), Ghana (13) and Namibia (13) account for 68% of international enrolments, with 32% (28) spread across Botswana, Eritrea, Kenya, Mauritius, Tanzania, Uganda and Zambia. Of the 344 enrolments with available data, the majority (92%; 318) were female.

Data regarding enrolments were analysed by year and educational programme (see Fig. 1). Programme team members advised that a maximum of 20 students could enrol on each of the two Postgraduate Diploma programmes annually, to comply with the terms of accreditation and limits on availability of clinical placements. In 2008, the first year that training was offered, 18 students were enrolled in total across the two programmes. Demand for training peaked in 2016 with 40 students enrolled. Enrolments declined in the following two years, reducing to 31 in 2018. The average (mean) number of students enrolled each year was 32.

Of the 337 enrolments for which data on source of fee payment was available, the majority (68%; 229) were employer-funded, 27% (90) received bursaries, 3% (10) were self-funded and 2% (8) were supported through mixed sources. Data shows a gradual decline in employer funded sponsorships and an increase in external bursaries sourced by the CNPDI. Of the 293 enrolments known to originate from hospital facilities, nine out of 10 are linked to public institutions. Specifically, 92% (221) of South African enrolments were shown to be public hospital employees.

Based on a field completion rate of 89%, 94% (293) of enrolments were found to be linked to 71 hospital sites. Seven educational institutions accounted for 4% (13) of student enrolments, with 2% (5) of nurses recorded as having resigned their jobs to study.

#### Demand for training

293 nurse enrolments could be linked to identifiable hospital facilities, of which 90% (264) were public hospitals and 5% (16) private institutions, sub-categorised into faith-based (5), non-profit (6) and for-profit (5) entities. In South Africa, 59% (141) of enrolments were concentrated around three sites in the Western and Eastern Cape (The Red Cross War Memorial Children’s Hospital: 101, Tygerberg Hospital: 24, Frontier Hospital: 16). The remainder were spread across 50 facilities in six of the country’s nine provinces. Other notable concentrations were in Malawi (Kamuzu Central Hospital: 11, Queen Elizabeth Central Hospital: 5), and Ghana (Komfo Anokye Teaching Hospital: 7).

Through member checking the programme team members were invited to consider the data showing fluctuations and recent decline in demand for training. The programme team considered that declining demand for training may reflect wider system challenges affecting the release of nurses for training, including employer financial constraints and facility-based staff shortages. The programme team attributed the decreased demand for training from Malawi, Kenya and Zambia to the deliberate strategies of educational institutions that partnered with CNPDI to establish their own in-country training programmes, which required upskilling for both specialist trained children’s nurses and nurse educators. This explanation was corroborated by further analysis of data (see ‘Graduates in education roles’ below). The programme team members also suggested that some hospital facilities which had been major drivers of training demand to meet clinical service needs (e.g. the Red Cross Children’s Hospital in South Africa) had achieved their staff training goals and subsequently reduced their demand for training. This explanation was not possible to verify within the scope of this study.

#### Course completions

Course completion records were analysed counting returning students only once, at their level of their highest qualification. Almost all students (94%) completed their educational programmes between January 2008 and December 2018 (318 out of a potential 340 graduations). 5% (18) of students (18) withdrew or failed to complete their studies. Programme staff reported that four students enrolled in 2017 and 2018 were expected to graduate in 2019 following extensions granted for personal or academic reasons. Across all students data revealed that 170 children’s nurses, 131 critical care children’s nurses and 11 advanced paediatric nurse practitioners had completed their training.

### Geographical mapping of graduates

**Fig. 3:**
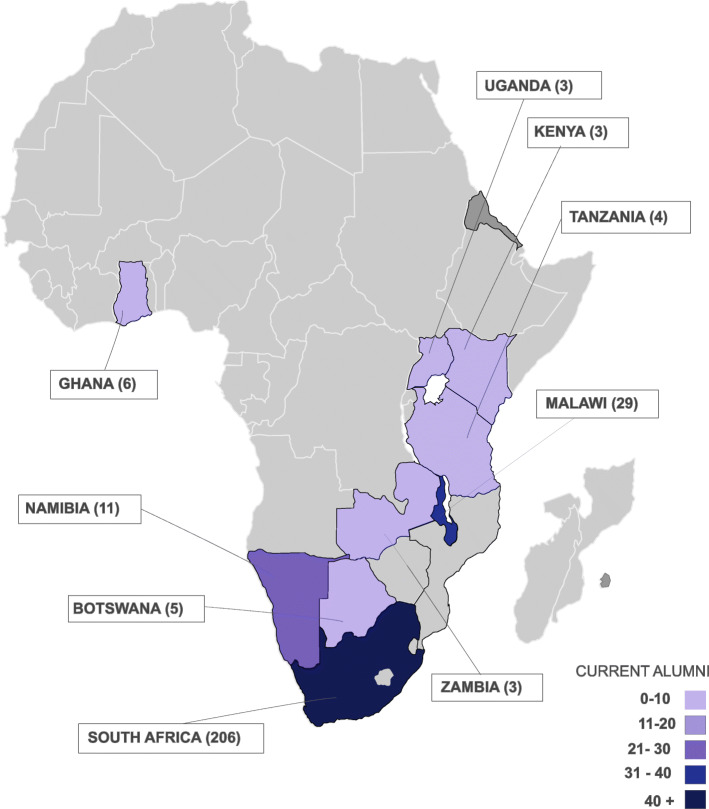
Distribution of graduates by country (2018)

Recorded information for graduates was analysed by country, region and facility, showing reported place of work and practice environment on graduation, to assist in understanding graduate mobility and retention. Available programme data included information obtained through a graduate follow up exercise undertaken by programme staff in 2018, which asked graduates to update their place of work. Information about place of work after graduation was available for 272 out of 312 graduates (field completion rate 87%). 99% (270) of graduates were shown to be working in Africa (see Fig. 3). Of the 248 where specific follow up data is available, 91% (226) continue to work at their original centre of employment. Available data shows minimal change in alumni situation, with just 9% (22) of graduates showing a change in circumstance, including movement into educational roles.

**Fig. 4:**
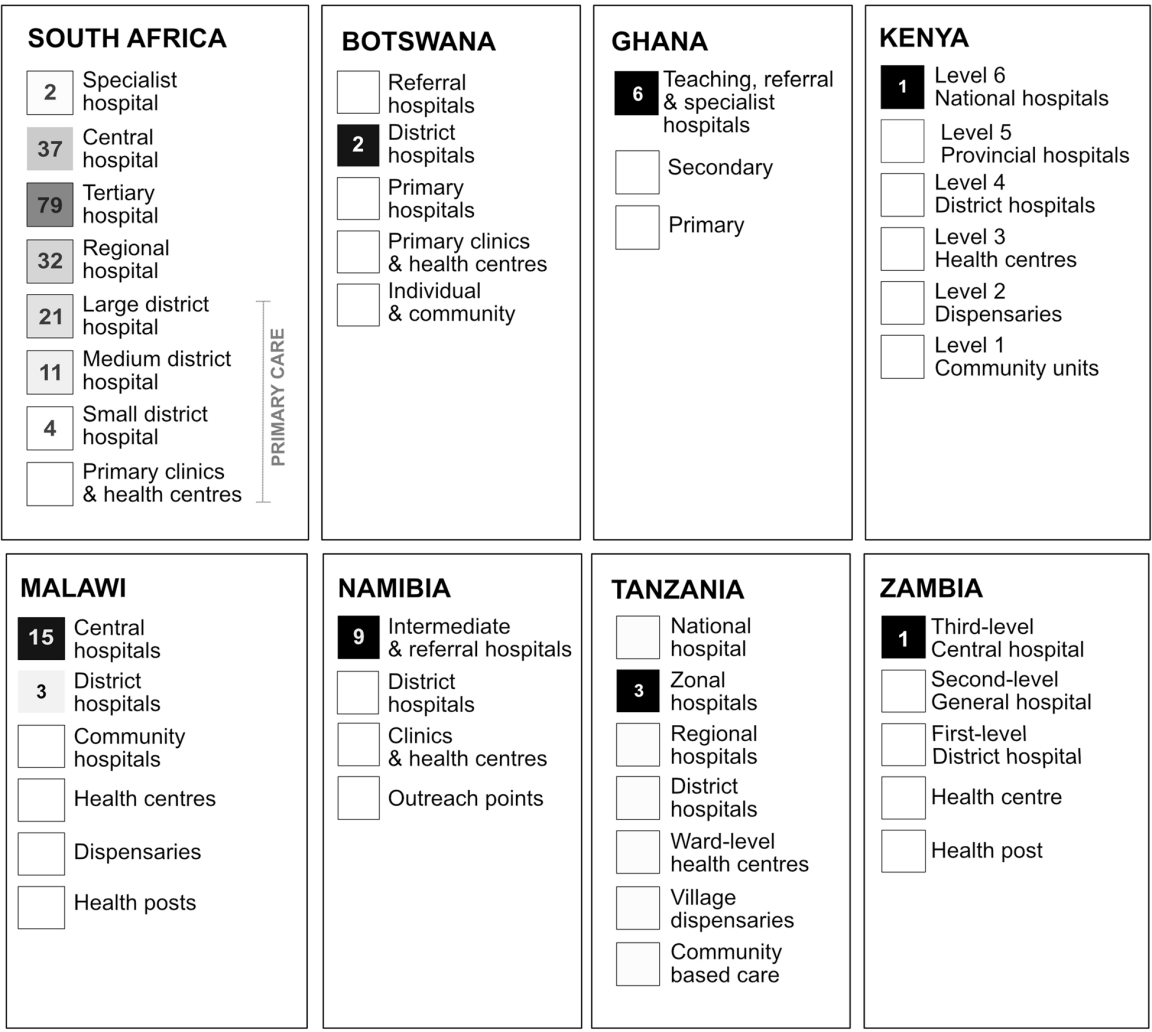
Known institution level of hospital-based graduates (2018)

The majority of graduates (238; 88%) practice at 68 different hospital facilities. Of these, 89% (212) work in public hospitals and 7% (16) in private facilities (for profit: 9, non-profit: 4, faith-based: 3, unknown type: 10). Analysis of facility type using information obtained from online public sources was carried out (see Fig. 4). Data shows the majority of alumni (84%; 190 out of 226) linked to Tier One or Two facilities (central and regional referral hospitals offering specialist inpatient paediatric services, sometimes called secondary and tertiary level care) providing high acuity and specialised care to children. This is particularly the case for international enrolments, where all graduates were found to be linked to Tier One or Two facilities and/or to education institutions affiliated to these facilities. In South Africa, 36 (19%) of alumni were found to be working at primary level sites. Reflecting enrolment data, most South African graduates were shown as based in the Western Cape with notable employment clusters at the Red Cross War Memorial Children’s Hospital (65), Tygerberg Hospital (25), and a further cluster at Frontier Hospital in the Eastern Cape (14). This distribution reflects the location of existing specialty and subspecialty paediatric facilities.

#### Graduates in education roles

Records suggest that at the end of 2018, 24 graduates were working in education roles, compared to 13 at the time of enrolment. Follow up data also showed 10 graduates moving from hospital to education environments on completion of training. Fifty percent of this shift happened in Malawi (5) with the rest spread across South Africa (3), Kenya (1) and Uganda (1). Through member checking with programme team members, it was established that more than half of these graduates (58%; 14) were reported to be working in institutions that offer (or have plans to offer) children’s nursing education programmes in South Africa, Botswana, Kenya, Malawi, Namibia and Zambia. This explanation was consistent with published data regarding children’s nursing training activity in the region [11] (North et al. 2018).

#### Explanations of graduate mobility

Member checking was also used to explore programme team members’ senses of why graduate mobility was so low. In response to the question: ‘Why have graduates chosen to remain in the public sector? Why have they not looked to sell their skills abroad?’, programme staff explained that the programme was intentionally designed to promote graduates’ return to practice in their home countries. South African students graduate with an educational qualification from the University of Cape Town which is registerable as an additional qualification with the South African Nursing Council. Graduates from other countries proceed to register the qualification with their relevant national nursing council. In this way, graduates are not immediately eligible to practice outside their home country. In addition, programme staff explained that nurses who received study leave and financial support from their employers would often be expected to enter into an agreement (locally known as a bond) to return to work for a specified period of time on completion of training. The findings from the follow up exercise suggested however that graduates were being retained in service beyond the period of the bond.

## Discussion

Analysis of data regarding student enrolment, demand for training, course completions and graduate employment offered encouraging insights in relation to training activity and throughput. Analysis of data in relation to the geographical mapping of graduates highlighted a strong concentration in Tier One and Two facilities, which is considered below in relation to the programme’s stated indirect goal of contributing to progress toward UHC.

### Contribution to workforce strengthening

Findings suggest that the programme is succeeding in meeting its stated direct goal of contributing to strengthening the capacity of the children’s nursing workforce in southern and eastern Africa, although the graduate outputs from this single programme are dwarfed by the scale of need. Accurate calculation of demand for training requires a comprehensive consideration of both supply and demand factors in context [38, 39], with fiscal capacity to meet the costs of training and re-absorb graduates into the workforce key. It was beyond the scope of this study to explore demand factors in more detail. However, given that the programme team reported that they were able to meet all demand for training over the period, with no international applicants meeting entry criteria ever turned away, it seems that demand for training is extremely low relative to global assessments of need [1-3].

### Retention of graduates

The findings in relation to the retention of graduates in-country by their original employers, and the lack of public-private drift, are encouraging. Again, the reasons for this were not explored. Mitigation of risk factors may be being achieved successfully through intentional programme design, as described by programme staff. Alternatively it is possible that the widely voiced concerns about skills migration are less of an issue than is sometimes assumed [15, 16]. The finding that both instances of international migration by graduates are linked to pursuit of doctoral studies aligns with the assertion of Munjanja et al. that migration frequently occurs in pursuit of education, not because of it [40].

### Course completions

The course completion rate of 94% is noteworthy in the context of the 30% average in-training attrition rate of health profession students within the Africa region [16]. Attrition rates for undergraduate nursing students in South African universities can also sit as high as 59% [41]. A study of South African postgraduate nursing students concluded that the most common reasons for failing to complete studies were a lack of institutional and social support; students’ inability to cope with rigorous academic demands; and difficult experiences connected to the cultural transformation of the teaching and learning environment [42]. The factors underpinning CNPDI’s high course completion rates were not explored by this study and may be worthy of further exploration as a successful model of nurse education.

### Training the trainers

An important finding in relation to longer-term sustainable change resulting from programme outputs is the evidence of South African and international graduates employed by education institutions on completion of training. Cominsky et al. have highlighted the crucial relationship between educational output and workforce development, with few courses delivering graduates with the capacity/aptitude to become nurse educators [21]. In contrast, programme data suggest the establishment of a growing cohort of children’s nurse educators. Set in the context of wider programme activity, this represents a direct contribution to eight new training programmes at institutions in Malawi, Kenya and Zambia.

### Graduate deployment in the context of universal health coverage

Geographic mapping of graduate locations and analysis of graduate employment by type and level of facility was instructive. A functioning child health system requires adequate staffing across community, primary, secondary and tertiary services [11, 43], to facilitate effective integrated service provision. Global [1-3, 44, 45] and local [46] strategies for achieving UHC envisage a role for specialist nurses across the care continuum, from primary and community level care to tertiary level facilities. The very strong concentration of graduates in secondary and tertiary level hospital facilities provides encouraging evidence that capacity is being built at specialist referral centres. This study found no evidence of a corresponding focus on primary and community settings, with fewer than one in five South African programme graduates and no international graduates going on to work in primary care and community level settings. Further research to understand the composition of the existing nursing workforce in primary and community care would be helpful in assessing future workforce development needs in the context of UHC.

### Strengths and limitations

It must be noted that the data only relate to graduates of the University of Cape Town’s children’s nursing educational programmes. The lack of available information about training activity at other institutions is a limitation. During the period of study the University of Cape Town was one of seven educational institutions in South Africa actively producing children’s nursing graduates. However, unique aspects of the programme include the University’s position as the only provider of critical care children’s nursing education in South Africa and one of only two providers in southern and eastern Africa. The University of Cape Town was also the South African institution which graduated the majority of international children’s nursing students from other African countries during the period of study [12, 31]. The data analysed therefore provide a reasonably comprehensive account of training outputs in relation to child critical care trained nurses, and child nursing students from southern and eastern African countries without their own in-country training programmes. An expanded follow up study to record graduate destinations for other training providers would be desirable.

The design of the study was influenced by pragmatic concerns common to many programme evaluation studies [32]. The study is likely to have been enriched by travel and interviewing if resources had been available, and further research involving face to face data collection and in-depth graduate follow up would be valuable.

This study is a retrospective evaluation and is only able to offer descriptive data. It is hoped however that the unique nature of the training intervention described, together with the regional significance of the outcomes, may still offer useful insights to those interested in building specialist nursing workforce capacity in Africa and other low-middle income countries.

## Conclusions

The children’s nursing education programmes at the University of Cape Town and the wider programmatic activities of CNPDI appear to be supporting the development of sustainable children’s nursing workforce capacity for the southern and east African region. Analysis of programme data suggests that a quantifiable and positive contribution has been made to the development of both the specialist clinical children’s nursing workforce in South Africa, Malawi, Namibia, Ghana, Botswana, Tanzania, Kenya, Zambia and Uganda, and also the specialist children’s nursing educator workforce, directly impacting the establishment of new training programmes in three African countries during the study period. The programme appears to have been successful in overcoming a number of known challenges associated with nurse education programmes. Further research to explore explanations for the very high rates of course completion and graduates retained in service would be useful.

Despite encouraging findings in relation to this single programme, training output for the region as a whole is still far from adequate to achieve a significant scale up of children’s nurses in a way that could meaningfully impact progress towards child-health related Sustainable Development Goals [4]. Given the evidence of a recent decline in demand for training places through the programme, mirrored by a decrease in financial support for trainees from public sector employers, we recommend urgent work by national ministries of health with global health assistance to clarify shared stakeholder visions for the specialist children’s nursing workforce in the context of UHC. We also advocate the development of costed plans for children’s nursing workforce development which balance need and affordability, prioritising the development of the specialist educator workforce and clinical learning hubs.

## Data Availability

The findings of this study are restricted for reasons of confidentiality and data protection under the terms of the consents obtained. Data were used under license for the current study and are not publicly available. The full report of this study including presentations of all anonymised analysed data is available in the Zenodo repository https://zenodo.org/record/3741318#.XorbSIgzZPY. The Theory of Change for the programme is available from: http://www.childnursingpractice.uct.ac.za/sites/default/files/image_tool/images/198/PDF/TheoryOf%20Change_Sep2018.pdf

## Abbreviations

CNPDI: Child Nurse Practice Development Initiative
HCRI: Humanitarian and Conflict Response Institute
MNCN: Master of Nursing in Child Nursing
NHWA: National Health Workforce Accounts
PGDip-CN: Post Graduate Diploma in Children’s Nursing
PGDip-CCCN: Post Graduate Diploma in Critical Care Children’s Nursing
UHC: Universal Health Coverage
WHO: World Health Organization
